# LONGITUDINAL RELATIONSHIPS OF DEPRESSIVE SYMPTOMS IN OLDER ADULTS WITH HYPERTENSION IN CHINA

**DOI:** 10.1093/geroni/igae098.2676

**Published:** 2024-12-31

**Authors:** Xiaomeng Wang, Zhi-wen Wang

**Affiliations:** Peking University, Beijing, Beijing, China (People’s Republic); Peking University Health Science Center, Beijing, Beijing, China (People’s Republic)

## Abstract

**Objectives:**

This study aimed to develop cross-lagged symptom networks and examine the longitudinal relationships of depressive symptoms among older adults with hypertension in China. Method: This study utilized data from the China Health and Retirement Longitudinal Study conducted in 2018 (T1) and 2020 (T2). The 10-item Center for Epidemiologic Studies Depression Scale (CES-D) was employed to measure depressive symptoms. A cross-lagged panel network (CLPN) analysis was applied to construct cross-lagged symptom networks for the entire sample as well as for males and females separately.

**Results:**

A total of 1937 participants were included in the final analysis. The strongest direct positive effect was observed from the item ‘D03: Felt Depressed’ to ‘D01: Bothered by Things’ (β = 0.13). For the full sample and the male, ‘D10: I Could Not Get on’ reported the largest value of out-strength. For female, ‘D03: Felt Depressed’ reported the largest value of out-strength (r = 1.350). ‘I Could Not Get on’ was the strongest predictor compared to the other nine depressive symptoms based on node centrality. Conclusions: Our study suggests that health care professionals should be particularly attentive when older adults with hypertension report difficulties in coping with daily activities, as well as feelings of diminished motivation and energy. Interventions based on motivations should be chosen with cation for older adults with hypertension in China.

